# TNF -308 G/A Polymorphism and Risk of Acne Vulgaris: A Meta-Analysis

**DOI:** 10.1371/journal.pone.0087806

**Published:** 2014-02-03

**Authors:** Jian-Kang Yang, Wen-Juan Wu, Jue Qi, Li He, Ya-Ping Zhang

**Affiliations:** 1 Laboratory for Conservation and Utilization of Bio-Resources & Key Laboratory for Microbial Resources of the Ministry of Education, Yunnan University, Kunming, Yunnan, China; 2 Department of Dermatology, First Affiliated Hospital of Kunming Medical University, and Institute of Dermatology & Venereology of Yunnan Province, Kunming, Yunnan, China; 3 State Key Laboratory of Genetic Resources and Evolution, and Yunnan Laboratory of Molecular Biology of Domestic Animals, Kunming Institute of Zoology, Chinese Academy of Sciences, Kunming, Yunnan, China; Tor Vergata University of Rome, Italy

## Abstract

**Background:**

The -308 G/A polymorphism in the tumor necrosis factor (*TNF*) gene has been implicated in the risk of acne vulgaris, but the results are inconclusive. The present meta-analysis aimed to investigate the overall association between the -308 G/A polymorphism and acne vulgaris risk.

**Methods:**

We searched in Pubmed, Embase, Web of Science and CNKI for studies evaluating the association between the -308 G/A gene polymorphism and acne vulgaris risk. Data were extracted and statistical analysis was performed using STATA 12.0 software.

**Results:**

A total of five publications involving 1553 subjects (728 acne vulgaris cases and 825 controls) were included in this meta-analysis. Combined analysis revealed a significant association between this polymorphism and acne vulgaris risk under recessive model (OR = 2.73, 95% CI: 1.37–5.44, *p* = 0.004 for AA *vs*. AG + GG). Subgroup analysis by ethnicity showed that the acne vulgaris risk associated with the -308 G/A gene polymorphism was significantly elevated among Caucasians under recessive model (OR = 2.34, 95% CI: 1.13–4.86, *p* = 0.023).

**Conclusion:**

This meta-analysis suggests that the -308 G/A polymorphism in the *TNF* gene contributes to acne vulgaris risk, especially in Caucasian populations. Further studies among different ethnicity populations are needed to validate these findings.

## Introduction

Acne vulgaris is a chronic inflammatory skin disease widely affecting adolescents and young adults [1]. The pathogenesis of acne vulgaris is a complex process in which several factors have been implicated, including hormonal effects, abnormal keratinocyte function, microbial components (Propionibacterium acnes), inflammation, environmental factors and genetics [2]–[4].

Inflammation plays one of the main roles in the development of acne vulgaris [5], [6]. A research in vivo reported that a marked increase for tumor necrosis factor (*TNF*) gene transcripts was observed in acne lesions [7]. TNF is one of the main pro-inflammatory cytokines that play a central role in initiating and regulating the cytokine cascade during an inflammatory response [8].

Several single nucleotide polymorphisms (SNPs) in the *TNF* gene promoter have been identified [9], [10], some of which may regulate *TNF* expression. One of these polymorphisms at position -308 (*TNF* -308 G/A) had been reported associated with regulation of *TNF* expression by, e.g., interfering with transcription factor binding sites or other regulatory elements [11].

A number of case-control studies were conducted to investigate the association of -308 G/A polymorphism in the *TNF* gene and the risk of acne vulgaris [12]–[17]. However, these studies reported conflicting results which may be due to the limitations in sample size and different ethnic populations in the corresponding investigation. Therefore, we perform present meta-analysis to systematically clarify the association between the *TNF* -308 G/A polymorphism and acne vulgaris risk based on all eligible case-control studies.

## Materials and Methods

### Literature Search

Two authors independently performed systematic searches in Pubmed, Embase, Web of Science and CNKI (China National Knowledge Infrastructure) databases to identify studies examining the association between the -308 G/A polymorphism in the *TNF* gene and acne vulgaris risk. A date limit of August 1, 2013 was applied. The search was performed without any restrictions on language. The search terms were as follows: “Acne vulgaris” or “Acne” in combination with “*TNF*” or “tumor necrosis factor”. The reference lists of identified studies and review articles were manually searched to find additional relevant publications.

### Study Selection

Studies were included in the meta-analysis if they satisfied the following inclusion criteria: (1) case-control studies focused on associations between *TNF* gene -308 G/A polymorphisms and acne vulgaris risk; (2) genotype frequencies were available for cases and controls; (3) the distribution of genotypes in the control group was consistent with Hardy-Weinberg equilibrium (HWE). (4) when publications involved the overlapping data sets, only the study with the largest number of participants was included. The supporting PRISMA checklist is available as Checklist S1.

### Data Extraction

Two authors independently extracted data from the included studies. The following data were extracted: the name of the first author, year of publication, country of origin, ethnicity, sample size, genotyping method and genotype frequencies in acne vulgaris cases and controls. In case of conflicting evaluations, disagreements were resolved through discussion between the authors.

### Statistical Analysis

The association between the *TNF* -308 G/A polymorphism and risk of acne vulgaris was assessed using odds ratios (ORs) and 95% confidence intervals (CIs). The significance of the pooled OR was determined using the Z-test and *p*<0.05 was considered statistically significant. We estimated the association based on four genetic models: allele model (A vs. G), dominant model (AA + AG vs. GG), recessive model (AA vs. AG + GG) and additive model (AA vs. GG).

To evaluate whether the association showed any ethnicity-specific effects, we analyzed the data for separate subgroups defined by ethnicity. Heterogeneity was evaluated using a *χ2*-based Q statistic and *I* ^2^ test, with *p*<0.10 or *I^2^*>50% considered statistically significant [18], [19]. When *p*≥0.10 or *I^2^*≤50%, the pooled OR was calculated using a fixed effect model (Mantel-Haenszel method); otherwise, a random effect model (DerSimonian Laird method) was used.

Publication bias was assessed using Begg's funnel plots and Egger's test [20]. Sensitivity analysis was performed by excluding individual studies and recalculating the results in order to assess the stability of the results. Pearson's *χ2* test was used to determine whether the observed frequencies of genotypes in control group conformed to the HWE [21]. All statistical tests were performed using STATA 12.0 software.

## Results

### Characteristics of included studies

**Figure 1:**
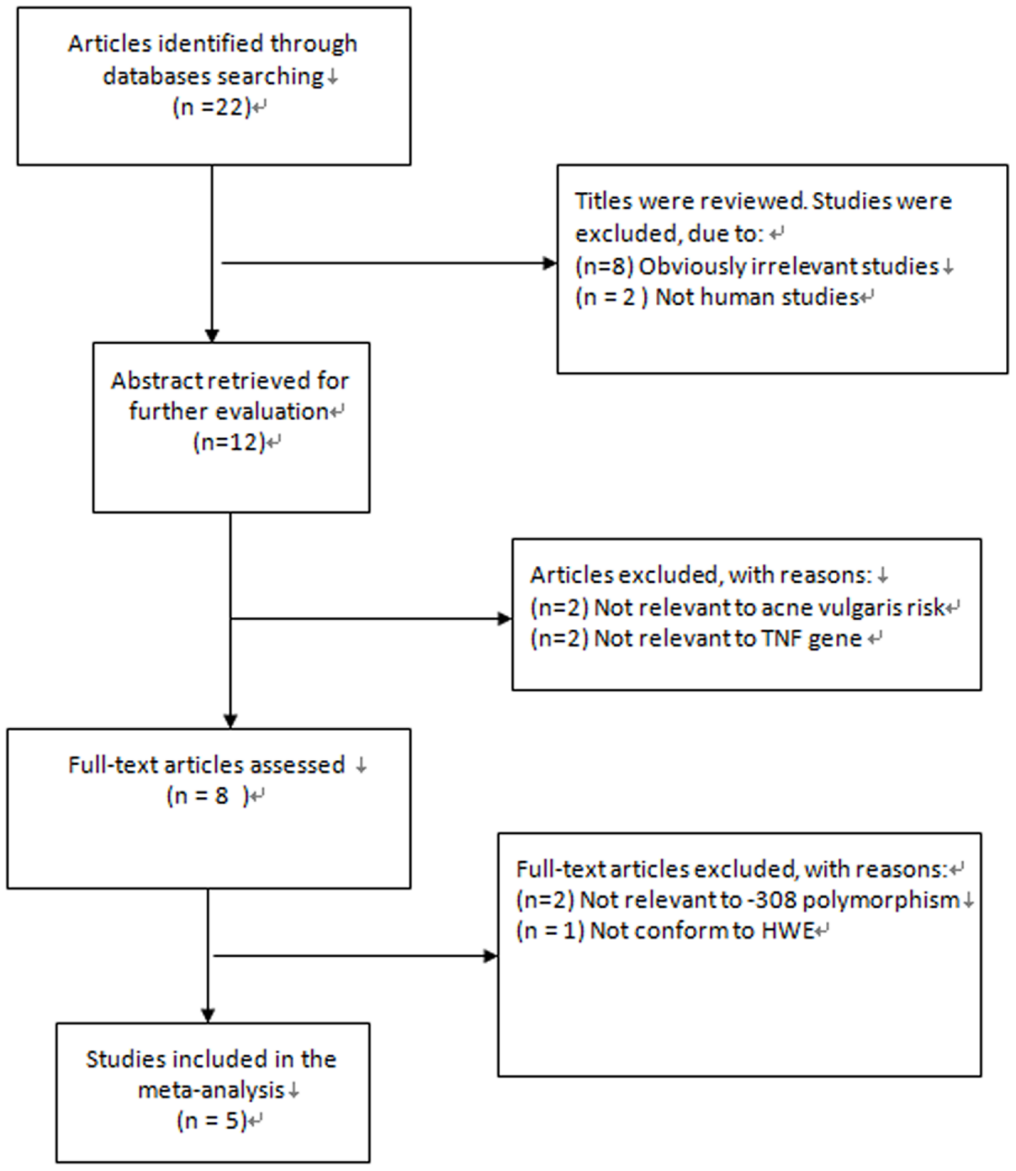
Flow chart of literature search and study selection.

The flow chart that displays the study selection process was shown in Figure 1. A total of five publications evaluating the association between the *TNF* -308 G/A polymorphism and acne vulgaris risk were included in the meta-analysis, involving 1553 subjects (728 acne vulgaris cases and 825 controls) [12]–[16]. One study was excluded because the distribution of genotypes in the control group was inconsistent with HWE [17]. Among the eligible five studies, four studies were performed in Caucasian populations and one study was in Asian population. The diagnosis of acne was based on a thorough physical examination by dermatologists. The clinical grade of acne was assessed mostly based on the Global Acne Grading System [22]. Acne patients were classified based on severity of the disease into three subgroups: mild, moderate, and severe acne. The control group was chosen from healthy individuals without any systemic and dermatologic diseases. In general, studies were relatively small, and the mean number of cases was 146 (range, 84–229) and the mean number of controls was 165 (range, 75–390). The characteristics of the included studies were summarized in Table 1 and Table 2.

**Table 1:** Characteristics of the five studies included in this meta-analysis.

Author	Year	Country	Ethnicity	Cases				Controls
				Mild (%)	Moderate (%)	Severe (%)	Total	
Baz K et al [12].	2008	Turkey	Caucasian	32 (28.3)	51 (45.1)	30 (26.6)	113	114
Sobjanek M et al [13].	2009	Poland	Caucasian	—	—	—	84	75
Szabo K et al [14].	2011	Hungary and Romania	Caucasian	29 (12.7)	156 (68.1)	44 (19.2)	229	126
Yu J et al [15].	2011	China	Asian	—	—	—	138	120
Al-Shobaili HA et al [16].	2012	Saudi Arabia	Caucasian	44 (26.8)	72 (43.9)	48 (29.3)	164	390

Note: - means that no data of acne severity in original paper.

**Table 2:** Distribution of *TNF* -308 G/A polymorphism in acne patients and control subjects.

Study	Allele				Genotype					
	Case		Control		Case			Control		
	A	G	A	G	AA	AG	GG	AA	AG	GG
Baz K et al [12].	51	175	15	213	4	43	66	0	15	99
Sobjanek M et al [13].	20	148	27	123	3	14	67	1	25	49
Szabo K et al [14].	80	378	32	220	4	72	153	1	30	95
Yu J et al [15].	62	214	24	216	10	42	86	1	22	97
Al-Shobaili HA et al [16].	63	265	166	614	10	43	111	12	142	236

### Meta-analysis results

**Figure 2:**
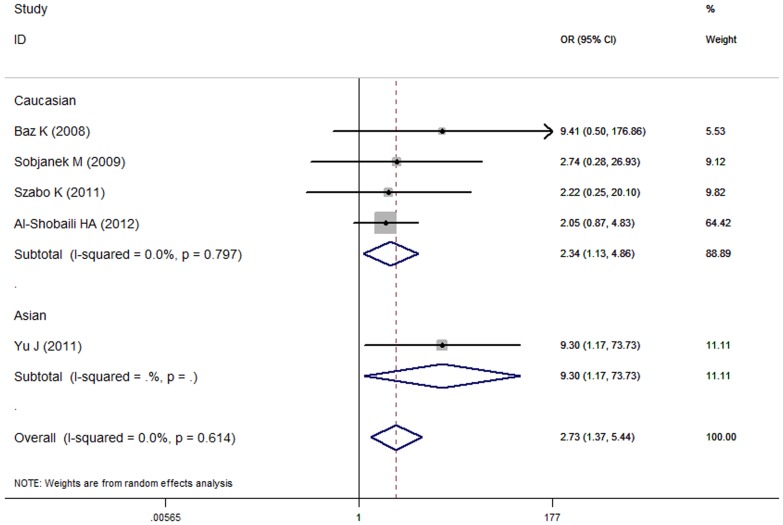
Meta-analysis to evaluate the association between the TNF -308 G/A polymorphism and acne vulgaris risk. Analysis were performed under recessive model (AA *vs*. AG + GG).

With significant between-study heterogeneity by *Q* test and *I^2^* test under the majority of genetic models (*I^2^*>50%), the analysis was conducted using random effect model. The meta-analysis results showed that *TNF* -308 G/A polymorphism was linked to the risk of acne vulgaris under recessive and additive models ( AA *vs.* AG + GG: OR = 2.73, 95%CI: 1.37–5.44, *p* = 0.004; AA *vs.* GG: OR = 2.67, 95%CI: 1.28–5.57, *p* = 0.009; respectively) (Figure 2). Results for these and other genetic models were summarized in Table 3.

**Table 3:** Meta-analysis of the association between *TNF* -308 G/A polymorphism and acne vulgaris risk.

	A vs. G		AA+AG vs. GG		AA vs. AG+GG		AA vs. GG	
	OR (95% CI)	*p*	OR (95% CI)	*p*	OR (95% CI)	*p*	OR (95% CI)	*p*
Total	1.52 (0.83–2.80)	0.180	1.44 (0.69–3.00)	0.327	**2.73 (1.37–5.44)**	**0.004**	**2.67 (1.28–5.57)**	**0.009**
Subgroup by Ethnicity								
Caucasian	1.33 (0.67–2.63)	0.417	1.25 (0.53–2.93)	0.606	**2.34 (1.13–4.86)**	**0.023**	**2.14 (1.02–4.47)**	**0.044**
Asian	**2.61 (1.57–4.33)**	**0.001**	**2.55 (1.44–4.51)**	**0.001**	**9.30 (1.17–73.73)**	**0.035**	**11.28 (1.42–89.93)**	**0.022**

Note: The bold values mean that their association is significant.

### Subgroup Analysis

In the subgroup analysis based on ethnicity, the results indicated that *TNF* -308 G/A polymorphism might significantly increase the risk of acne vulgaris among Caucasian populations (recessive model: AA *vs.* AG + GG, OR = 2.34, 95%CI: 1.13–4.86, *p* = 0.023; additive model: AA *vs.* GG, OR = 2.14, 95% CI: 1.02–4.47, *p* = 0.044; respectively). *TNF* -308 G/A polymorphism might show significant association with the increased risk of acne vulgaris in all genetic models among Asian populations (all *p*<0.05), but not enough reliability was established due to the result from a single study [15] (Table 3).

### Sensitivity Analysis and Publication Bias

Sensitivity analysis were performed by sequential omission of individual studies for all subjects and subgroups. The corresponding pooled ORs were not significantly altered in all subjects and subgroups (data not shown). The results of sensitivity analysis indicated the stability of our results. Begg's funnel plot and Egger's test were used to assess publication bias. The shape of the funnel plots in all the genetic models seemed symmetrical, indicating that there were no evidences for obvious publication bias. Further, Egger's test provided similar results that there were no statistically significant publication bias in all genetic models (all *p*>0.05).

## Discussion

The *TNF* gene is located on chromosome 6 (6p21.3) between HLA-B and DR within the class III region of the major histocompatibility complex [23]. There are several polymorphisms in the promoter region of the *TNF* gene (−863, −857, −850, −575, −375, −308, −274, −238, −237, −162) [9], [10]. The most common polymorphisms is in the promoter at position *−308*. The polymorphism may affect cytokine production [11], [24], [25]. To date, several studies have been carried out to identify whether *TNF* -308 G/A polymorphism was associated with acne vulgaris risk. However, the conclusion was controversial. To the best of our knowledge, our article is the first meta-analysis evaluating the association between *TNF* -308 G/A polymorphism and acne vulgaris risk. We performed the present meta-analysis of eligible five independent case-control studies, including 728 cases and 825 controls. We were able to provide a more complete picture of the role of *TNF* -308 G/A polymorphisms in acne vulgaris risk, as comparing with that published in individual studies.

>When all the eligible studies were pooled into the meta-analysis, the results showed that -308 G/A polymorphism was associated with the risk of acne vulgaris under the recessive model, with AA homozygote at higher risk of acne vulgaris than AG heterozygote and GG homozygote. In addition, we performed a stratified analysis based on ethnicity. The results showed that -308 G/A polymorphism might increase the risk of acne vulgaris among Caucasian population under recessive model, and that the homozygotic AA genotype may be a risk factor for acne vulgaris. We also found the -308 G/A polymorphism to be a risk factor for acne vulgaris among Asians, but the result in Asian population was not reliable enough due to the estimation from a single study [15]. More studies are needed to perform in Asian population. Our finding that the -308 G/A polymorphism was a genetic risk factor for acne vulgaris both in Caucasians and Asians, suggested no population-specific genetic difference in acne vulgaris pathogenesis.

*TNF* -308 G/A polymorphism have been examined in several autoimmune and inflammatory diseases, such as psoriasis, lepromatous leprosy and systemic lupus erythematosus [26]–[28]. However, the results were not consistent, mainly due to differences of the studied populations, or insufficient sample size. *TNF* -308 A allele had shown to be a stronger transcriptional activator than the common *TNF* -308 G allele in vitro and patients with *TNF* -308 GA heterozygosity had increased TNF production [11], [29].

The findings in this meta-analysis should be interpreted with caution because of several limitations. First, a relatively small number of studies were included and the sample sizes were still relatively small, which may not provide sufficient power to estimate the association between *TNF* -308 G/A polymorphism and acne vulgaris risk. Second, the included publications were limited to Caucasian and Asian populations, so future work should examine other populations. Third, although no obvious publication bias was identified, potential bias cannot be completely ruled out. Fourth, there had been some differences in severity of cases between the included studies (Table 1), in which mild, moderate and severe acne occupied different proportion, although the diffrences were not statistically significant. Nonetheless, it was well acknowledged that many other factors, such as gene-environment interactions may affect the risk of acne vulgaris.

## Conclusions

To the best of our knowledge, this is the first meta-analysis to assess the relationship between the *TNF* -308 G/A polymorphism and acne vulgaris risk. Our results suggest that the -308 G/A polymorphism may be a potential risk factor for acne vulgaris, especially in Caucasian population. However, further studies are still needed to warrant and validate the association between *TNF* -308 G/A gene polymorphism with acne vulgaris risk.

## Supporting Information

Checklist S1 **PRISMA Checklist.**
(DOC)
